# Changes in Electron Paramagnetic Resonance Parameters Caused by Addition of Amphotericin B to *Cladosporium cladosporioides* Melanin and DOPA-Melanin—Free Radical Studies

**DOI:** 10.3390/ijms25179571

**Published:** 2024-09-04

**Authors:** Magdalena Zdybel, Barbara Pilawa, Teresa Witoszyńska, Dorota Wrześniok

**Affiliations:** 1 Department of Biophysics, Faculty of Pharmaceutical Sciences in Sosnowiec, Medical University of Silesia in Katowice, Jedności 8, 41-200 Sosnowiec, Poland; bpilawa@sum.edu.pl; 2 Department of Pharmaceutical Chemistry, Faculty of Pharmaceutical Sciences in Sosnowiec, Medical University of Silesia in Katowice, Jagiellońska 4, 41-200 Sosnowiec, Poland; chemlek@sum.edu.pl (T.W.); dwrzesniok@sum.edu.pl (D.W.)

**Keywords:** amphotericin B, melanin, *Cladosporium cladosporioides*, free radicals, EPR spectroscopy

## Abstract

*Cladosporium cladosporioides* are the pigmented soil fungi containing melanin. The aim of this work was to determine the influence of amphotericin B on free radicals in the natural melanin isolated from pigmented fungi *Cladosporium cladosporioides* and to compare it with the effect in synthetic DOPA-melanin. Electron paramagnetic resonance (EPR) spectra were measured at X-band (9.3 GHz) with microwave power in the range of 2.2–70 mW. Amplitudes, integral intensities, linewidths of the EPR spectra, and g factors, were analyzed. The concentrations of free radicals in the tested melanin samples were determined. Microwave saturation of EPR lines indicates the presence of pheomelanin in addition to eumelanin in *Cladosporium cladosporioides*. o-Semiquinone free radicals in concentrations ~10^20^ [spin/g] exist in the tested melanin samples and in their complexes with amphotericin B. Changes in concentrations of free radicals in the examined synthetic and natural melanin point out their participation in the formation of amphotericin B binding to melanin. A different influence of amphotericin B on free radical concentration in *Cladosporium cladosporioides* melanin and in DOPA-melanin may be caused by the occurrence of pheomelanin in addition to eumelanin in *Cladosporium cladosporioides*. The advanced spectral analysis in the wide range of microwave powers made it possible to compare changes in the free radical systems of different melanin polymers. This study is important for knowledge about the role of free radicals in the interactions of melanin with drugs.

## 1. Introduction

Amphotericin B belongs to the polyene group and is used as an antifungal drug [1,2,3,4,5]. Amphotericin B is effective, among others, against *Candida albicans*, *Candida krusei*, *Candida tropicalis*, *Candida parapsilosis* [2], *Cryptococcus* spp. [1,2,4], *Fusarium* spp., *Rhizopus* spp., and *Histoplasma* spp. [1,4]. Antifungal treatment by amphotericin B may be recommended in coronavirus disease 2019 (COVID-19) patients with secondary fungal co-infection [6]. The binding of amphotericin B to ergosterol in the cell membrane in fungi resulted in cell killing [1,2,5,7]. Amphotericin B forms free radicals and leads to oxidative damage in cells. This drug stimulates phagocytic cells during fungal infection [7].

Melanin polymers occur in the human and animal organisms [8,9,10,11,12,13]. Melanin plays a major role in protecting the skin against ultraviolet damage [8,9]. The important role of melanin in melanoma was proved [8,10,11,12,13]. Melanin is also found in plants [14].

Fourier-transform infrared (FTIR), Raman, ultraviolet–visible (UV–Vis), solid-state nuclear magnetic resonance (ssNMR), and matrix-assisted laser desorption ionization mass spectrometry (MALDI-TOF MS) spectroscopic studies pointed out the existence of melanin in *Cladosporium cladosporioides* [15]. It has been chemically demonstrated that amphotericin B has an affinity for both synthetic and natural melanin polymers [16]. This drug forms complexes with the model eumelanin–DOPA-melanin and with the melanin isolated from *Cladosporium cladosporioides* mycelium [16]. The higher concentrations of amphotericin B and the longer incubation time resulted in an increase in the amount of the drug bound to melanin.

The inspiration to perform this research was information about free radicals in melanin and the possibility of examining them by the use of electron paramagnetic resonance (EPR) spectroscopy. The diamagnetic molecules were not examined. A spectroscopic method was used that allowed for the study of paramagnetic units. We examined only paramagnetic centers through the absorption of microwave radiation in a magnetic field. Iconic EPR research has identified o-semiquinone free radicals in these polymers [17]. Contemporary research confirmed the participation of free radicals in interactions of melanin with metal ions [18,19]. The aim of this work was to determine the influence of amphotericin B on free radicals in natural melanin isolated from the pigmented fungi *Cladosporium cladosporioides* and to compare it with the effect in synthetic DOPA-melanin.

The shape of the EPR spectra depends on the type of melanin [20,21,22]. EPR spectra of eumelanins are measured as single lines, while a complex shape is observed for pheomelanins [20]. The EPR spectra constituting a composition of lines of eumelanin and pheomelanin were observed for *Cladosporium cladosporioides* [21]. EPR spectroscopy with several frequencies of microwaves (S-, X-, and Q-band) was proposed in the study of natural melanin from a *Streptomyces cyaneofuscatus* strain [22]. The complex character of the EPR spectra of melanin from the actinobacteria strain was presented. Eumelanin and pheomelanin were distinguished by EPR measurements and by magnetic relaxation [22]. Different behavior of melanin from different samples results from the differences in the chemical structure of these polymers. Free radicals always have unpaired electrons localized on the oxygen atom, but the spin–lattice relaxation may differ because these processes result from interactions between unpaired electrons and the diamagnetic molecular lattice.

In our studies, free radicals in *Cladosporium cladosporioides* melanin were examined by the EPR method. The changes in the EPR spectra of this melanin biopolymer were used to characterize the effect of amphotericin B on free radicals in the fungi melanin. The practical importance of the research lies in its evaluation possibilities of assessing the role of free radicals in interactions of amphotericin B in organisms during treatment.

## 2. Results and Discussion

**Figure 1:**
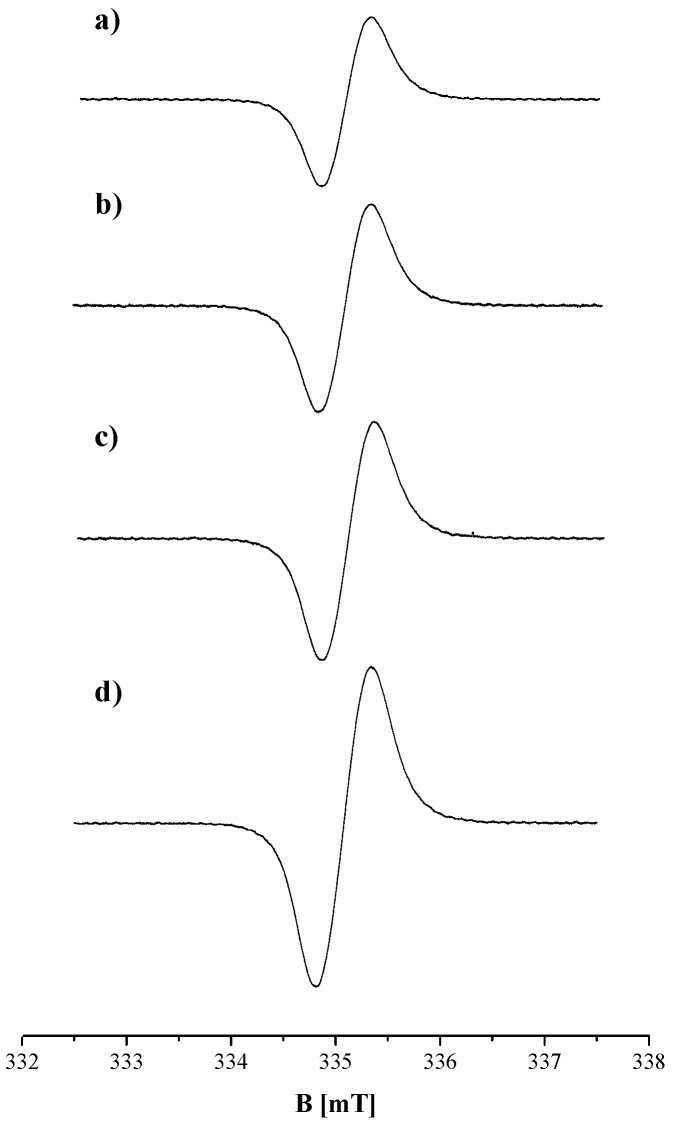
EPR spectra of (**a**) DOPA-melanin and its complexes with amphotericin B at concentrations of (**b**) 1.8 µg/cm^3^, (**c**) 4.5 µg/cm^3^, and (**d**) 8.1 µg/cm^3^. B is the magnetic induction. The presented EPR spectra of the melanin samples were measured with a microwave power of 2.2 mW.

**Figure 2:**
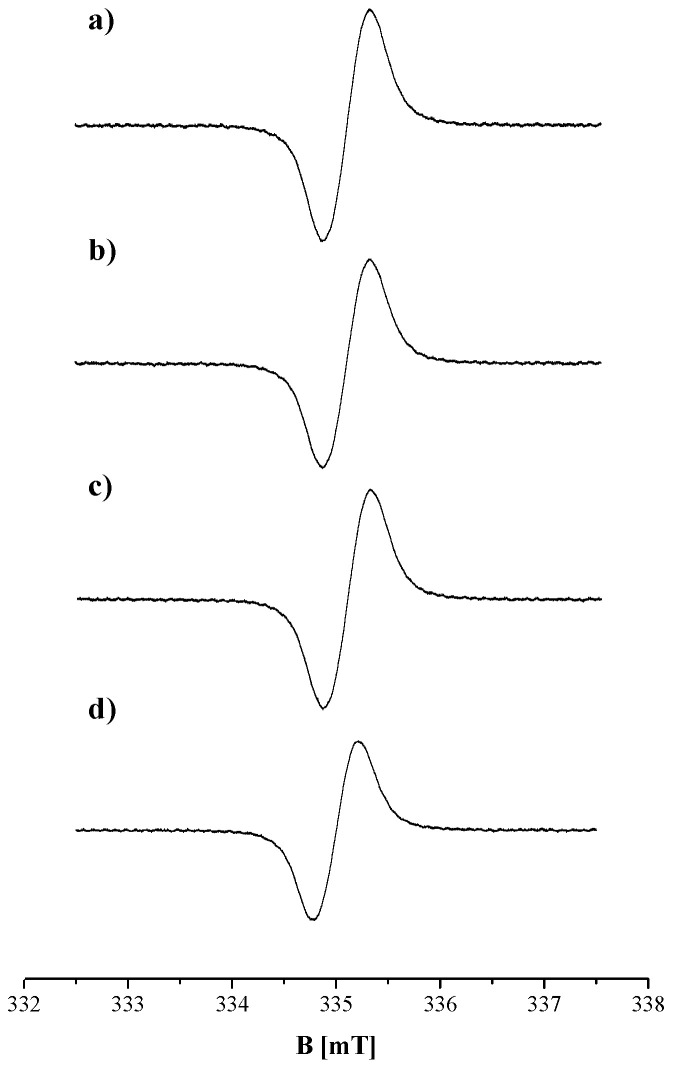
EPR spectra of (**a**) melanin isolated from *Cladosporium cladosporioides* and its complexes with amphotericin B at concentrations of (**b**) 1.8 µg/cm^3^, (**c**) 4.5 µg/cm^3^, and (**d**) 8.1 µg/cm^3^. B is the magnetic induction. The presented EPR spectra of the melanin samples were measured with a microwave power of 2.2 mW.

EPR spectra were obtained for DOPA-melanin, melanin isolated from *Cladosporium cladosporioides*, complexes of DOPA-melanin with amphotericin B, and complexes of melanin isolated from *Cladosporium cladosporioides* with amphotericin B. The EPR spectra, measured with a microwave power of 2.2 mW, of DOPA-melanin and complexes of DOPA-melanin with amphotericin B in concentrations of this drug: 1.8 µg/cm^3^, 4.5 µg/cm^3^, and 8.1 µg/cm^3^, are presented in Figure 1, respectively. The EPR spectra of melanin isolated from *Cladosporium cladosporioides* and its complexes with amphotericin B at concentrations of 1.8 µg/cm^3^, 4.5 µg/cm^3^, and 8.1 µg/cm^3^, measured with a microwave power of 2.2 mW, are presented in Figure 2. The measured EPR signal of melanin originates from o-semiquinone free radicals. EPR spectroscopy allows free radicals to be distinguished from the other paramagnetic centers. The decisive parameter in this matter is the g factor, which depends on the localization of unpaired electrons in free radical molecules. Free radicals have a g factor near 2. EPR studies of melanin have demonstrated that these polymers contain o-semiquinone free radicals with unpaired electrons located on oxygen atoms. The EPR studies by other authors [17,18,19,20] and our earlier EPR studies of melanin [21,23,24] provided the values of g factors. Characteristic g factors in the range of 2.0038–2.0040 were obtained. These radicals are stable and can be detected using continuous-wave EPR. The more reactive free radical species are short-lived and, therefore, are not detected by the classical method. The reactive short-living radicals react with the other molecules, and they become diamagnetic. The diamagnetic species are not monitored by EPR spectroscopy.

The EPR spectra of *Cladosporium cladosporioides* melanin are asymmetrical lines. The asymmetry results from the superposition of lines originating from the two types of melanin that we met in *Cladosporium cladosporioides*. Eumelanin mainly exists in these pigmented fungi and is responsible for the first component line in the EPR spectrum. The EPR spectrum of natural eumelanin is similar to the EPR spectrum of synthetic DOPA-melanin. The EPR spectra of these two melanins are single lines. Additionally, pheomelanin with an EPR line revealing the unresolved hyperfine structure is present in *Cladosporium cladosporioides.* The asymmetry of the EPR spectrum of *Cladosporium cladosporioides* melanin and its complexes with amphotericin B results from adding two lines with different amplitudes (A), different linewidths (ΔB_pp_), and shifted g values. These two lines come from two different melanin polymers, and their sum gives the experimentally measured spectrum. The shift in the maximum amplitude (A) due to the effect of microwave power on the amplitude of DOPA-melanin and *Cladosporium cladosporioides* melanin samples confirmed the presence of two different structures in the tested natural melanin. The complex shapes of the EPR spectra of *Cladosporium cladosporioides* were obtained earlier [21]. EPR signals of eumelanin and pheomelanin were also observed in the EPR spectra of the other pigmented fungi, such as *Cladosporium herbarum* [23]. Lines of eumelanin were observed for the melanin synthesized by *Bacillus weihenstephanensis* soil strains [24]. EPR lines of pheomelanin-like pigment were detected in the mycelium of *Plenodomus biglobosus* [25]. EPR spectroscopy is a tool for examining the type of melanin in biological samples [20,21,22,23,24,25,26,27,28,29].

Table 1 includes the spectral parameters for the tested DOPA-melanin samples: g factors, amplitudes (A), integral intensities (I), and linewidths (ΔB_pp_). The same parameters of the EPR spectra of the tested *Cladosporium cladosporioides* melanin samples are shown in Table 2. EPR lines of all tested melanin samples reveal the same values of g factor (2.0040). The obtained g value (2.0040) (Table 1 and Table 2) and the earlier results [17,20,23,27] indicate that o-semiquinone free radicals exist in *Cladosporium cladosporioides* melanin and its complexes with amphotericin B. This value is characteristic of o-semiquinone free radicals in melanin [18,23,24,27,28,29]. Similar linewidths were obtained for DOPA-melanin (ΔB_pp_: 0.48 mT) (Table 1) and natural *Cladosporium cladosporioides* melanin (ΔB_pp_: 0.45 mT) (Table 2). Taking into account the accuracy of the measurements (±0.02 mT), the bounding of amphotericin B to DOPA-melanin (Table 1) and *Cladosporium cladosporioides* melanin (Table 2) does not change the linewidths (ΔB_pp_) of the EPR lines of these melanin polymers. The measured lines were broad, which indicates strong dipolar interactions in the samples [30].

The higher amplitudes (A) have the EPR lines of the complexes of DOPA-melanin with amphotericin B (Table 1). The lower amplitudes (A) have the EPR lines of the complexes of *Cladosporium cladosporioides* melanin with amphotericin B (Table 2). Integral intensities (I) of the EPR spectra of DOPA-melanin complexes with amphotericin B are higher than integral intensity (I) of the EPR line of DOPA-melanin (Table 1). Integral intensities (I) of the EPR spectra of the complexes of *Cladosporium cladosporioides* melanin with amphotericin B are lower than the integral intensity (I) of the EPR line of this natural melanin (Table 2). The mentioned differences between integral intensities (I) of EPR lines are the most visible for the melanin complexes with amphotericin B in concentration 8.1 [µg/cm^3^].

The increase in amplitude (A) and integral intensity (I) with higher drug concentrations in the complexes of DOPA-melanin with amphotericin B (Table 1) indicates that the free radical concentration increases with the addition of more drug to this melanin. The decrease in amplitude (A) and integral intensity (I) after the addition of amphotericin B to *Cladosporium cladosporioides* melanin (Table 2) indicates a reduction in free radicals in this natural melanin due to the tested drug.

**Figure 3:**
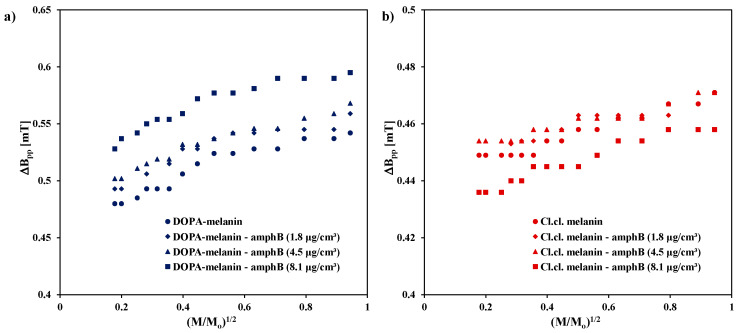
The influence of microwave power on the linewidth (ΔB_pp_) of the EPR spectra of (**a**) DOPA-melanin and its complexes with amphotericin B at concentrations of 1.8 µg/cm^3^, 4.5 µg/cm^3^, and 8.1 µg/cm^3^, and (**b**) melanin isolated from *Cladosporium cladosporioides* and its complexes with amphotericin B at the same concentrations. M_o_—the total microwave power produced by klystron (70 mW); M—microwave power used during the measurement of the EPR spectrum.

Linewidths (ΔB_pp_) of the EPR spectra of DOPA-melanin, melanin isolated from *Cladosporium cladosporioides*, and complexes of these two types of melanin with amphotericin B increase with increasing microwave power. The influence of microwave power on the linewidth (ΔB_pp_) of the EPR spectra of DOPA-melanin, its complexes with amphotericin B, *Cladosporium cladosporioides* melanin, and its complexes with amphotericin B at the tested drug concentrations is shown in Figure 3. The broadening of EPR lines with increasing microwave power was observed independent of the concentration of amphotericin B in the melanin complexes. All the EPR lines are homogeneously broadened. The EPR lines of DOPA-melanin, *Cladosporium cladosporioides* melanin, and their complexes with amphotericin B broaden with increasing microwave power because of the quantum phenomena. Magnetic interactions are responsible for the line broadening in homogenous systems [30].

**Figure 4:**
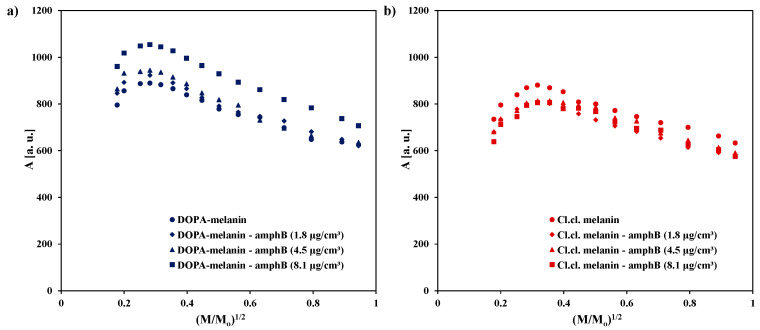
The influence of microwave power on the amplitude (A) of the EPR spectra of (**a**) DOPA-melanin and its complexes with amphotericin B at concentrations of 1.8 µg/cm^3^, 4.5 µg/cm^3^, and 8.1 µg/cm^3^, and (**b**) melanin isolated from *Cladosporium cladosporioides* and its complexes with amphotericin B at the same concentrations. M_o_—the total microwave power produced by klystron (70 mW); M—microwave power used during the measurement of the EPR spectrum.

The influence of microwave power on amplitudes (A) of the EPR spectra of DOPA-melanin and complexes of DOPA-melanin with amphotericin B in different concentrations is shown in Figure 4a. In our studies, we used different microwave powers ranging from 2.2 to 70 mW because microwave power is important in the examination of magnetic interactions in melanin polymers. It was checked whether amphotericin B affects spin–lattice relaxation in melanin. The changes in the amplitude of the EPR line of melanin with increasing microwave power allow for the determination of the speed of spin–lattice relaxation. For the fast spin–lattice relaxation processes, the amplitude of the EPR line reaches its maximum at higher microwave powers than for the slow processes. The similar character of the changes in amplitudes (A) as a function of microwave power used during measurements is visible for DOPA-melanin and for all DOPA-melanin–amphotericin B complexes. The amplitudes (A) of the detected EPR lines increase with increasing microwave power, reach the maximum, and then decrease with further increases in microwave power. The influence of microwave power on the amplitudes (A) of the EPR spectra of melanin isolated from *Cladosporium cladosporioides* and complexes of *Cladosporium cladosporioides* melanin with amphotericin B in the three used concentrations of this drug is shown in Figure 4b. Just like in the case of the DOPA-melanin samples, the amplitudes (A) of the EPR lines of *Cladosporium cladosporioides* melanin and its complexes with amphotericin B increase with increasing microwave power, and after reaching the maximum, they decrease with a further increase in microwave power. Microwave saturation of EPR lines at low microwave powers indicates the occurrence of slow spin–lattice relaxation processes in the tested melanin samples.

**Figure 5:**
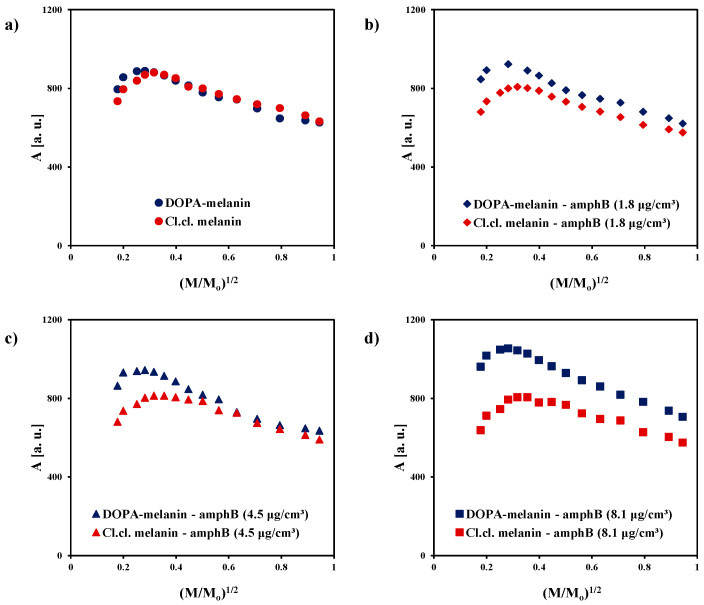
Comparison of the changes in amplitudes (A) of the EPR lines with increasing microwave power for (**a**) DOPA-melanin and melanin isolated from *Cladosporium cladosporioides*, (**b**) complexes of DOPA-melanin and *Cladosporium cladosporioides* melanin with amphotericin B at a drug concentration of 1.8 µg/cm^3^, (**c**) complexes of DOPA-melanin and *Cladosporium cladosporioides* melanin with amphotericin B at a drug concentration of 4.5 µg/cm^3^, and (**d**) complexes of DOPA-melanin and *Cladosporium cladosporioides* melanin with amphotericin B at a drug concentration of 8.1 µg/cm^3^. M_o_—the total microwave power produced by klystron (70 mW); M—microwave power used during the measurement of the EPR spectrum.

To compare the microwave saturation of the EPR lines of the DOPA-melanin samples and the *Cladosporium cladosporioides* melanin samples, the changes in their amplitudes (A) with increasing microwave power are shown together in Figure 5. The EPR lines of the *Cladosporium cladosporioides* melanin and its complexes with amphotericin B saturate at a higher microwave power than the EPR lines of DOPA-melanin and DOPA-melanin complexes with amphotericin B. The amplitudes (A) of the *Cladosporium cladosporioides* samples reach the maximum later than amplitudes (A) of DOPA-melanin samples. The relatively faster spin–lattice relaxation processes occur in *Cladosporium cladosporioides* melanin and its complexes with amphotericin B than in DOPA-melanin and its complexes with amphotericin B. For the faster spin–lattice relaxation processes, the reversal of energy levels occurs at the higher microwave powers [30]. The differences in relaxation processes in DOPA-melanin as synthetic eumelanin and in melanin from *Cladosporium cladosporioides* as the complex system containing both eumelanin and pheomelanin were expected. Different microwave saturation of EPR spectra was observed for eu- and pheomelanin [17,20,26,27].

**Figure 6:**
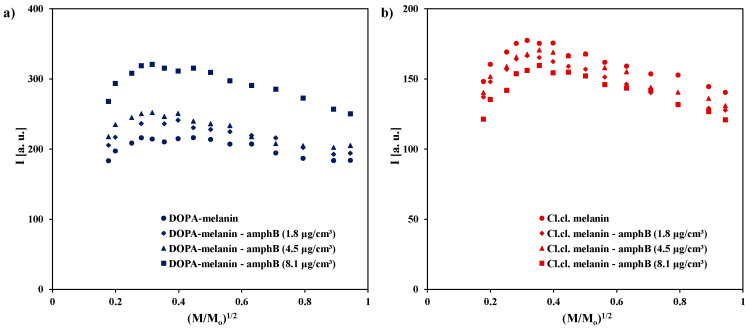
The influence of microwave power on the integral intensity (I) of the EPR spectra of (**a**) DOPA-melanin and its complexes with amphotericin B at concentrations of 1.8 µg/cm^3^, 4.5 µg/cm^3^, and 8.1 µg/cm^3^, and (**b**) melanin isolated from *Cladosporium cladosporioides* and its complexes with amphotericin B at the same concentrations. M_o_—the total microwave power produced by klystron (70 mW); M—microwave power used during the measurement of the EPR spectrum.

The integral intensities (I) of the EPR lines of DOPA-melanin and DOPA-melanin complexes with amphotericin B (Figure 6a), as well as melanin isolated from *Cladosporium cladosporioides* and its complexes with amphotericin B (Figure 6b), depend on microwave power in the same way as the amplitudes (A) (Figure 4). The integral intensities (I) initially increase with increasing microwave power, and then after reaching maximum values, they become smaller and smaller for higher powers. The maxima of the integral intensities (I) are similar for melanins and for the complexes of amphotericin B with the right type of melanin.

**Figure 7:**
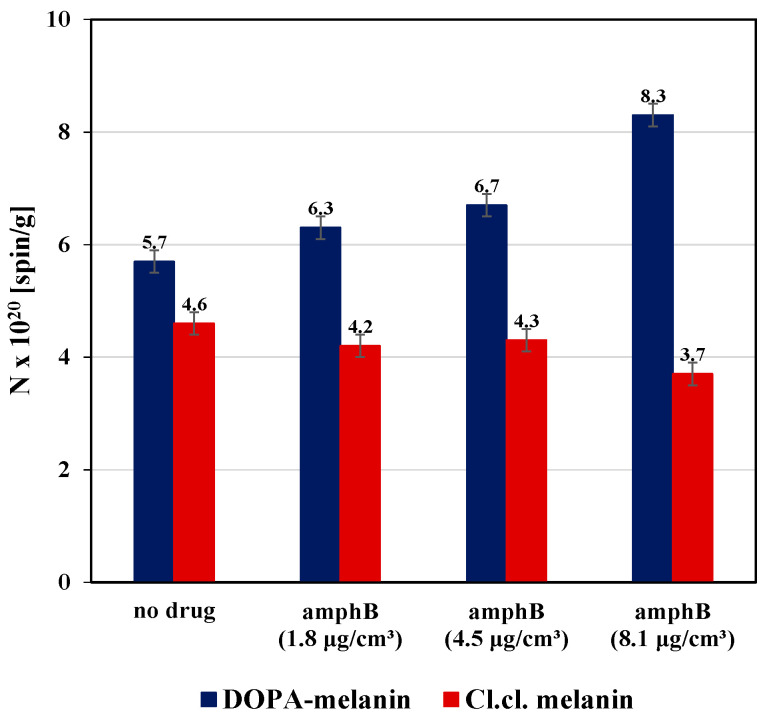
Comparison of free radical concentrations (N) in DOPA-melanin, melanin isolated from *Cladosporium cladosporioides,* and complexes of these melanins with amphotericin B at concentrations of 1.8 µg/cm^3^, 4.5 µg/cm^3^, and 8.1 µg/cm^3^.

The high concentrations (N) of free radicals characterize the tested primary melanin polymers and their complexes. Concentration values are on the order of 10^20^ [spin/g]. For comparison, free radical concentrations in the other melanins were 2.6 × 10^18^ [spin/g] (melanin from *Hermetia illucens*) [31], 3.2 × 10^19^ [spin/g] (melanin from *Sepia officinalis*) [32], and 1.7–3.3 × 10^22^ [spin/g] (melanin from *Bacillus weihenstephanensis)* [24]. The concentrations (N) of free radicals in DOPA-melanin, melanin isolated from *Cladosporium cladosporioides,* and their complexes with amphotericin B at different concentrations of the tested drug (1.8 µg/cm^3^, 4.5 µg/cm^3^, and 8.1 µg/cm^3^) are compared in the bar chart presented in Figure 7. In Figure 7, the maximum errors of the measurement values are marked.

The concentration (N) of free radicals in melanin isolated from *Cladosporium cladosporioides* is lower than in DOPA-melanin (Figure 7). The concentration (N) of free radicals in complexes of *Cladosporium cladosporioides* melanin with amphotericin B is lower compared to that in DOPA-melanin complexes with amphotericin B. A different effect of amphotericin B binding was also noticed for *Cladosporium cladosporioides* melanin and for DOPA-melanin. The binding of amphotericin B to melanin from *Cladosporium cladosporioides* leads to a reduction in the concentration (N) of free radicals in the melanin, whereas the binding of amphotericin B causes an increase in the free radical concentration (N) in DOPA-melanin. For both melanin polymers, only weak changes in (N) values were observed after complexing with lower concentrations of amphotericin B (1.8 µg/cm^3^ and 4.5 µg/cm^3^). The use of amphotericin B at a concentration of 8.1 µg/cm^3^ caused a significant increase in free radical concentrations (N) in DOPA-melanin and a significant decrease in free radical concentrations (N) in *Cladosporium cladosporioides* melanin.

The effect of the increase in the free radical concentration in DOPA-melanin during the binding of amphotericin B has not been thoroughly explained so far. A similar effect was observed after binding of the diamagnetic metals, for example, Zn^2+^, to melanin [8,17,18]. Probably, the increase in the EPR signal of free radicals may be the result of changes in relaxation processes in unpaired electrons of free radicals in the melanin [8,17,18]. The explanation of this effect requires further research.

Free radical formation in DOPA-melanin after complexation by amphotericin B (Figure 7) may have negative consequences. DOPA-melanin is the model eumelanin, and eumelanin exists in nature. The formation of free radicals may result from the stronger chemical activity of this polymer. It can be assumed that a stronger reaction with oxygen and metal ions will take place.

The changes in the parameters of EPR lines of DOPA-melanin and *Cladosporium cladosporioides* melanin after the binding of amphotericin B indicate the participation of free radicals in binding this drug to melanin. Changes in EPR spectra were also observed for the other drugs during binding to melanin [27,33]. The modifications in free radicals caused by amphotericin B may result in changes in the treatment of diseases caused by fungi. This study confirmed the usefulness of EPR spectroscopy in the examination of interactions of amphotericin B with free radicals of melanin. Because of the role of free radicals [34,35,36,37,38], the application of EPR spectroscopy to test free radicals is important from the viewpoint of normal physiological functions in organisms and human diseases.

The pigmented fungi *Cladosporium* spp. that show the presence of melanin biopolymers in their cell walls [39] are allergens and can cause infections such as phaeohyphomycosis and chromoblastomycosis [40,41,42]. Flucytosine and amphotericin B are used in these infections with pigmented fungi *Cladosporium* spp. Amphotericin B, which belongs to polyene macrolide antibiotics, is used in most cases of mycoses [42,43]. Amphotericin B offers the highest chance of cure in disseminated candidiasis, cryptococcosis, and aspergillosis. Amphotericin B is used with flucytosine in cryptococcosis, organ candidiasis, in endocarditis caused by fungi, and in infections with fungi, including *Cladosporium cladosporioides* [16,43].

The chemical structure of melanin contains quinone groups, hydroquinone, semiquinone, aromatic rings, hydroxyl groups, and stable o-semiquinone free radicals [16,27]. Melanin has protective functions due to its ability to absorb light and thermal energy and to bind metal ions, proteins, pesticides, pollutants, and drugs [16,21,27,39,44]. The protective properties of the melanin polymer in relation to mycelium influence the interactions of fungal melanin with drugs [16]. These interactions may change the effectiveness of the drugs bound to melanin. The performed spectroscopic studies have expanded existing knowledge about free radicals in melanin from *Cladosporium cladosporioides* and its complexes with the antifungal drug amphotericin B.

## 3. Materials and Methods

### 3.1. Materials

Synthetic DOPA-melanin, natural melanin isolated from pigmented soil fungi *Cladosporium cladosporioides*, and complexes of these two types of melanin with amphotericin B were studied.

L-3,4-Dihydroxyphenylalanine (L-DOPA) was purchased from Sigma-Aldrich Inc. (St. Louis, MO, USA). DOPA-melanin was obtained by oxidative polymerization of L-DOPA in 0.067 M phosphate buffer (pH 8.0) for 48 h, according to the Binns method [45].

Soil fungi of the *Cladosporium cladosporioides* species were obtained from the natural environment of two locations—No. 1 and No. 10 [46]—in the Karkonosze Mountains from the Institute of Ecology, Dziekanów Leśny, Łomianki, and the isolated mycelium was transferred to the Department of Chemistry and Drug Analysis [21,44,47,48,49,50,51]. *Cladosporium cladosporioides* fungi were cultured at a temperature of 26–28 °C for 14 days. The standard medium containing glucose (20 g), yeast extract (10 g), peptone (10 g), and bidistilled water (ad 1 dm^3^) was used. The media were adjusted to pH 7.0 by adding sodium hydroxide solution. Then, the mycelium was filtered, washed with bidistilled water, and dried to a constant weight. In order to obtain melanin from *Cladosporium cladosporioides*, dry mycelium was degreased with ethyl ether, hydrolyzed in 6 M HCl at 110 °C for 24 h to remove the protein, and then washed with bidistilled water [52]. The obtained insoluble melanin was degreased with acetone and dried to a constant weight.

Complexes of amphotericin B with synthetic DOPA-melanin and melanin isolated from mycelium *Cladosporium cladosporioides* were obtained by incubating samples of 5 mg each with 5 mL of a drug solution. Amphotericin B solubilized (catalog number A9528; Sigma-Aldrich Inc. (St. Louis, MO, USA)) was dissolved in 5% glucose to the final concentrations 1.8, 4.5, and 8.1 μg/cm^3^. Amphotericin B concentrations were selected experimentally and described previously in a paper [16] on amphotericin B binding to melanin to cover the range of therapeutic concentrations. It should be mentioned that the reacting system of melanin and amphotericin B is complex. Amphotericin B solution was prepared in 5% glucose and then mixed with melanin. Glucose without magnetic moments does not absorb microwaves in magnetic fields. It is expected that glucose, being a diamagnetic molecule without EPR signals, does not affect the EPR spectra of melanin. The incubation of all the samples was conducted at room temperature for 90 min. After incubation, the suspensions were filtered and dried to a constant weight. The control samples were prepared simultaneously with the tested samples but without the drug.

### 3.2. EPR Measurements and Analysis

EPR spectroscopy can be used to examine the properties and concentrations of free radicals and to determine the antiradical potency of the compounds [20,30,53,54].

The EPR spectra of melanin samples were obtained by an X-band (9.3 GHz) EPR spectrometer of Radiopan Firm (Poznań, Poland). The fast numerical data acquisition system Rapid Scan Unit with software produced by Jagmar Firm (Kraków, Poland) and LabView 8.5 (National Instruments Corporation, Austin, TX, USA) were used during the measurements and analysis. Magnetic modulation was 100 kHz. The microwave frequency was detected by the MCM 101 recorder of EPRAD Firm (Poznań, Poland). The magnetic field meter of EPRAD Firm (Poznań, Poland) provided information about the magnetic induction of the field produced by the electromagnet.

EPR spectra were measured as the first derivative of microwave absorption in the wide range of microwave power from 2.2 mW to 70 mW. g factors, linewidths (ΔB_pp_), amplitudes (A), and integral intensities (I) of EPR lines were analyzed.

The g factor was calculated directly using the equation of the electron paramagnetic resonance condition [30]:

g = hν/μ_B_B_r_ (1)

where h—Planck constant, ν—microwave frequency, μ_B_—Bohr magneton, and B_r_—resonance magnetic induction. The g factor was used to determine the type of free radicals in biological systems and polymers [20,21,22,23,24,27,32,33,55,56,57,58,59,60].

Linewidths (ΔB_pp_) of the EPR lines depend on magnetic interactions in the tested structures with unpaired electrons [30,61,62]. Amplitudes (A) and integral intensities (I) of EPR lines are important parameters in the quantitative research of free radicals. Amplitude (A) and integral intensity (I) increase with increasing free radical content in the samples [30]. Integral intensity (I), as the area under the absorption curve [30], was obtained by double integration of the first derivative lines.

The free radical concentration (N) in the tested melanin and melanin complexes with amphotericin B was determined by the use of ultramarine as the paramagnetic reference. Ultramarine contains the stabile paramagnetic centers [63,64]. EPR lines of the examined samples and ultramarine were measured at the low microwave power of 2.2 mW to avoid microwave saturation of the spectra. Integral intensities of EPR lines of the tested melanin samples (I) and ultramarine (I_u_) were compared. The EPR lines of the ruby crystal, which was permanently placed in the resonance cavity, were measured. The concentration (N) of free radicals in the melanin samples was calculated from the following formula [30,63,64,65]:

N = n_u_[(W_u_A_u_)/I_u_][I/(WAm)] (2)

where n_u_—number of paramagnetic centers in ultramarine (the reference); W, W_u_—receiver gains for sample and ultramarine, respectively; A, A_u_—amplitudes of ruby signal for the sample and ultramarine, respectively; I, I_u_—integral intensities for the sample and ultramarine, respectively; and m—mass of the sample.

The tests were repeated three times, and the values of the parameters were averaged. Measurement errors were determined by the total differential method. The measured parameters were functions of several variables. According to the total differential method, the error Δf for multivariable functions f(x_1_, x_2_, …, x_n_) is expressed by the following pattern [66]:

Δf = [δf(x_1_, x_2_, …, x_n_)/δx_1_]•|Δx_1_| + [δf(x_1_, x_2_, …, x_n_)/δx_2_]•|Δx_2_| + … + [δf(x_1_, x_2_, …, x_n_)/δx_n_]•|Δx_n_| (3)

where

- δf(x_1_, x_2_, …, x_n_)/δx_1,_ δf(x_1_, x_2_, …, x_n_)/δx_2_ and δf(x_1_, x_2_, …, x_n_)/δx_n_ are the partial derivatives of functions f(x_1_, x_2_, …, x_n_) over variables x_1_, x_2_, and x_n_;
- |Δx_1_|, |Δx_2_| and |Δx_n_| are the mean maximum errors of the relevant parameters x_1_, x_2_, and x_n_, respectively.

The spectroscopic programs of Jagmar Firm (Kraków, Poland) and Origin (OriginLab, Northampton, MA, USA) were used.

Measurement accuracies for the tested parameters and values were ±0.0002 for the g factor, ±0.02 [mT] for the linewidth (ΔB_pp_), ±0.01 [a. u.] for the amplitude (A), ±0.02 [a. u.] for the integral intensity (I), and ±0.2 × 10^20^ [spin/g] for the free radical concentration (N). Changes in parameters smaller than the maximal errors were not considered in the discussion.

## 4. Conclusions

This study pointed out that the EPR lines of melanin isolated from *Cladosporium cladosporioides* and its complexes with amphotericin B saturate at a greater microwave power than the EPR lines of DOPA-melanin (the model eumelanin) and DOPA-melanin complexes with amphotericin B, which may result from the presence of pheomelanin in addition to eumelanin in *Cladosporium cladosporioides*. It was found that o-semiquinone free radicals ~10^20^ [spin/g] exist in DOPA-melanin, *Cladosporium cladosporioides* melanin, and their complexes with amphotericin B. Interactions of amphotericin B with DOPA-melanin and *Cladosporium cladosporioides* melanin change the concentrations of free radicals in these polymers, but the order of magnitude remains unchanged (~10^20^ spin/g). The free radical concentration in DOPA-melanin complexes with amphotericin B (8.1 µg/cm^3^) is 1.5 times greater than its value in DOPA-melanin, whereas it is 1.2 times smaller in complexes of melanin from *Cladosporium cladosporioides* compared to the originally tested fungal melanin. Changes in the concentrations of free radicals in the examined synthetic and natural melanin point out their participation in the formation of amphotericin B binding to melanin. A different influence of amphotericin B on the concentration of free radicals in melanin isolated from *Cladosporium cladosporioides* and DOPA-melanin can be explained by the presence of pheomelanin in addition to eumelanin in *Cladosporium cladosporioides*, so probably eumelanin and pheomelanin are involved in amphotericin B binding to *Cladosporium cladosporioides* melanin.

## Figures and Tables

**Table 1:** g factors, linewidths (ΔB_pp_), amplitudes (A), and integral intensities (I) of EPR spectra of DOPA-melanin and its complexes with amphotericin B at concentrations of 1.8 µg/cm^3^, 4.5 µg/cm^3^, and 8.1 µg/cm^3^. EPR spectra were measured with a microwave power of 2.2 mW.

Sample	g ± 0.0002	ΔB_pp_ [mT] ± 0.02	A [a. u.] ± 0.01	I [a. u.] ± 0.02
DOPA-melanin	2.0039	0.48	795.65	183.32
DOPA-melanin–amphB (1.8 μg/cm^3^)	2.0039	0.49	846.15	205.66
DOPA-melanin–amphB (4.5 μg/cm^3^)	2.0039	0.50	864.41	217.83
DOPA-melanin–amphB (8.1 μg/cm^3^)	2.0040	0.53	961.11	267.94

**Table 2:** g factors, linewidths (ΔB_pp_), amplitudes (A), and integral intensities (I) of EPR spectra of melanin isolated from *Cladosporium cladosporioides* and its complexes with amphotericin B at concentrations of 1.8 µg/cm^3^, 4.5 µg/cm^3^, and 8.1 µg/cm^3^. EPR spectra were measured with a microwave power of 2.2 mW.

Sample	g ± 0.0002	ΔB_pp_ [mT] ± 0.02	A [a. u.] ± 0.01	I [a. u.] ± 0.02
Cl.cl. melanin	2.0039	0.45	734.78	148.13
Cl.cl. melanin–amphB (1.8 μg/cm^3^)	2.0040	0.45	680.00	137.09
Cl.cl. melanin–amphB (4.5 μg/cm^3^)	2.0040	0.45	680.77	140.32
Cl.cl. melanin–amphB (8.1 μg/cm^3^)	2.0038	0.44	638.00	121.28

## Data Availability

The EPR data obtained in this study are available upon request from the authors.
